# A rare case report of a young male patient presented with a pericardial hydatid cyst

**DOI:** 10.1093/jscr/rjaf355

**Published:** 2025-10-16

**Authors:** Laith Daraghmeh, Malak Elaiwi, Yara Quzmar, Ghassan Baloushi, Hasan Salman, Mahmoud Hasan, Abdelkarim Adas

**Affiliations:** Department of Thoracic Surgery, An-Najah National University Hospital, Asira Street, PO Box 7, Hai Asira, District of Nablus, Nablus 23861, Palestine; Department of Medicine, Faculty of Medicine and Health Sciences, An-Najah National University, Nablus, Palestine; Rafidia Surgical Hospital, Rafidia Street, PO Box 696, Nablus 44839, Palestine; Faculty of Medicine, Al-Quds University, Abu Dis Main Campus, PO Box 89, East Jerusalem 51000, Palestine; Department of Thoracic Surgery, Al-Razi Hospital, Al-Kroum Street, Downtown, Jenin 15538, Palestine; Department of Thoracic Surgery, Al-Razi Hospital, Al-Kroum Street, Downtown, Jenin 15538, Palestine; Department of Thoracic Surgery, Al-Razi Hospital, Al-Kroum Street, Downtown, Jenin 15538, Palestine

**Keywords:** hydatid cyst, pericardium, sternotomy, pericardial cyst, cardiac echinococcosis, case report

## Abstract

In cardiac echinococcosis, the pericardium is rarely affected. In this report, we present an uncommon case of a pericardial hydatid cyst in a 24-year-old male patient who presented with a clinical picture of abdominal pain, vomiting, and shortness of breath. After he was admitted, he underwent investigations that included a cardiac echocardiography. He had a resection of that cyst via a midline sternotomy. His condition improved, and he was discharged home. We believe it is important to describe this case due to the remarkable rarity of this entity and the morbidity associated with the delay in diagnosis.

## Introduction

Humans acquire echinococcosis when they become infected with taeniid cestodes belonging to the Echinococcus genus. Although six species have been identified, four constitute a threat to public health [1]. The organs that are most frequently affected by cardiac echinococcosis are the lungs and liver, but it can also affect the heart and other organs; in fact, cardiac echinococcosis accounts for 0.5%–2% of cases [2].

This case report describes the extremely uncommon position of the hydatid cyst in the heart—the pericardium is even more unusual. The patient was admitted and managed at a private hospital. This case report has been reported in line with the SCARE Criteria [3].

## Case report

A 24-year-old male with no significant medical history presented with a 4-day history of abdominal pain, distension, recurrent vomiting, shortness of breath, weakness, and loss of appetite. He appeared ill with yellow discoloration of the eyes, elevated jugular venous pressure, muffled heart sounds, diffuse abdominal tenderness, and shifting dullness. His vital signs showed a fast heart rate of 124 beats per minute, high blood pressure of 155/90 millimeters of mercury, and an oxygen saturation of 95% while receiving 4 L per minute of oxygen through a nasal cannula.

**Figure 1:**
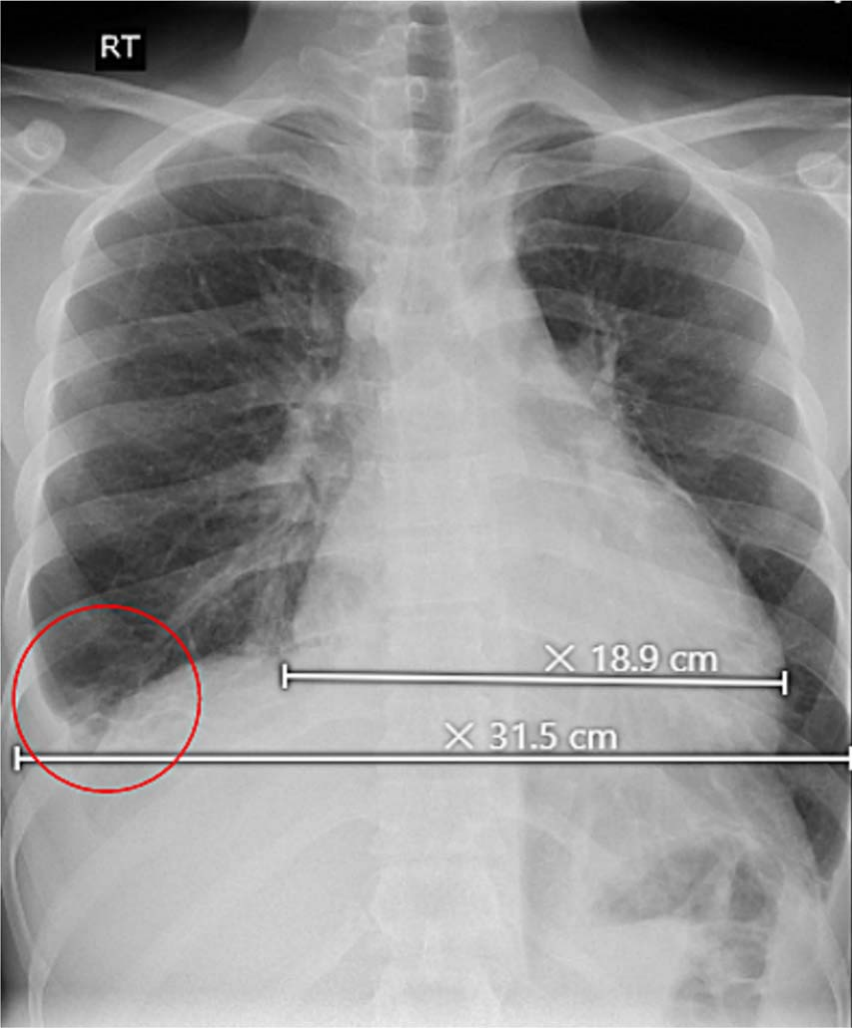
Upright chest X-ray, revealed an increase in cardiothoracic ratio with a minimal right-sided pleural effusion (circle).

Initial investigations revealed abnormal liver enzymes and elevated inflammatory markers (Table 1). A chest X-ray showed a minimal right-sided pleural effusion and an increased heart-to-chest ratio (Fig. 1). Abdominal ultrasound showed an enlarged liver measuring 18.2 cm, an irregular liver capsule, a mildly dilated portal vein of 1.4 cm, and fluid in the abdominal cavity, suggesting liver congestion. Analysis of the abdominal fluid showed a serum-to-ascitic albumin gradient >1.1, indicating a fluid buildup due to increased pressure in the blood vessels of the liver (Table 2).

**Table 1:** Initial laboratory results upon current admission

Parameter	Result	Reference range
White blood cell count	16.1	4–9 k/μl
Hemoglobin	13.5	13.7–17.2 g/dl
Platelets	230	140–450 k/μl
Amylase, Serum	1700	28–100 U/L
Aspartate aminotransferase (AST)	1114	15–40 U/L
Alanine aminotransferase (ALT)	1700	10–33 U/L
Alkaline phosphatase (ALP)	100	44 to 147 IU/L
Total serum bilirubin (TSB)	2.3	0.1 o 1.2 mg/dL
Direct serum bilirubin (DSB)	1.4	<0.3 mg/dl
Sodium	133	135–155 mEq/L
Potassium	4.3	3.5–4.8 mEq/L
Chloride	98	98–107 mEq/L
Creatinine	0.9	0.7–1.2 mg/dl
Prothrombin time	43	11–14 s
International normalized ratio	3.2	0.8–1.2
Activated partial thromboplastin time	40	25–40 s

**Table 2:** Peritoneal fluid analysis result

Parameter	Result	Reference range
Color	Yellow	
Appearance	Clear	
Protein	2.9	< 3 g/dl
Glucose	106	50–150 mg/dl
Red blood cell count (RBCs)	20	Normally absent
Total cell count	1700	0–300 cells/mcl
White blood cell count (WBCs)	30	<500 cells per μl)
Lactate dehydrogenase (LDH)	169	<225 units per L

**Figure 2:**
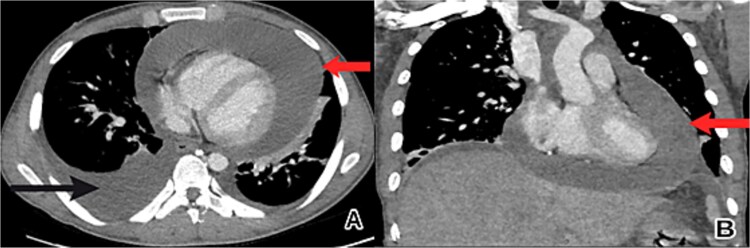
Chest CT scan showed a large pericardial effusion. (A) An axial view revealed a pericardial effusion (indicated by the upper arrow) and a right-sided pleural effusion (lower arrow). (B) A coronal view revealed a pericardial effusion (arrow) with small right heart chambers.

**Figure 3:**
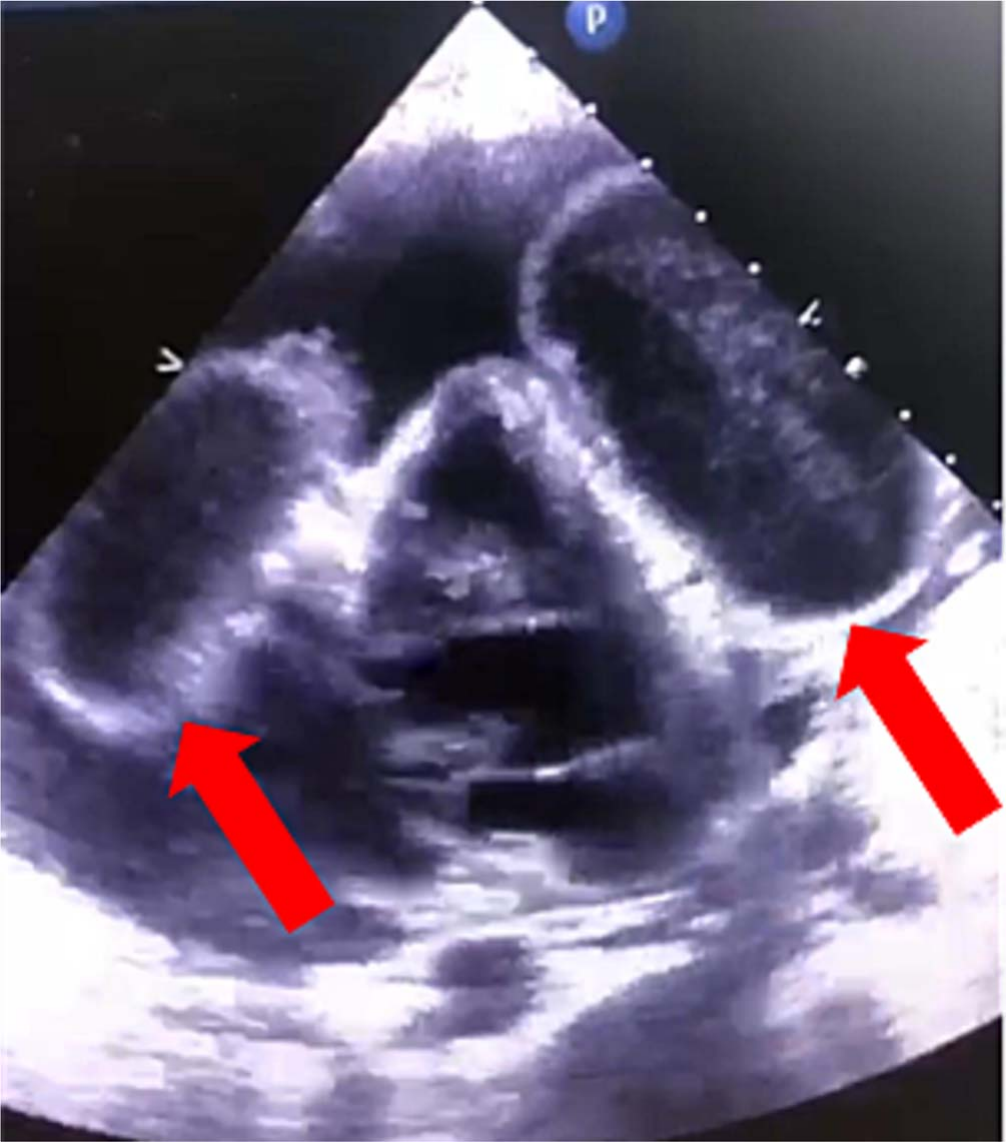
Cardiac echocardiography showed that the right ventricle was compressed by an oval-shaped large intrapericardial structure representing the cysts (arrows).

The patient’s condition worsened, developing a high fever, rapid breathing, and low blood pressure, leading to admission to the intensive care unit and treatment with medications to raise blood pressure. A full infection workup was negative. A computed tomography (CT) scan of the chest showed a large pericardial effusion measuring 5.5 cm in thickness and a small right heart chamber (Fig. 2). An echocardiogram revealed a normal left ventricle with an ejection fraction of 60%, but the right ventricle was compressed by a large oval-shaped structure inside the pericardial sac. The pericardial effusion measured 4.6 cm in thickness, and the inferior vena cava was dilated at 2.7 cm, with no heart valve regurgitation or blood clots inside the heart (Fig. 3).

**Figure 4:**
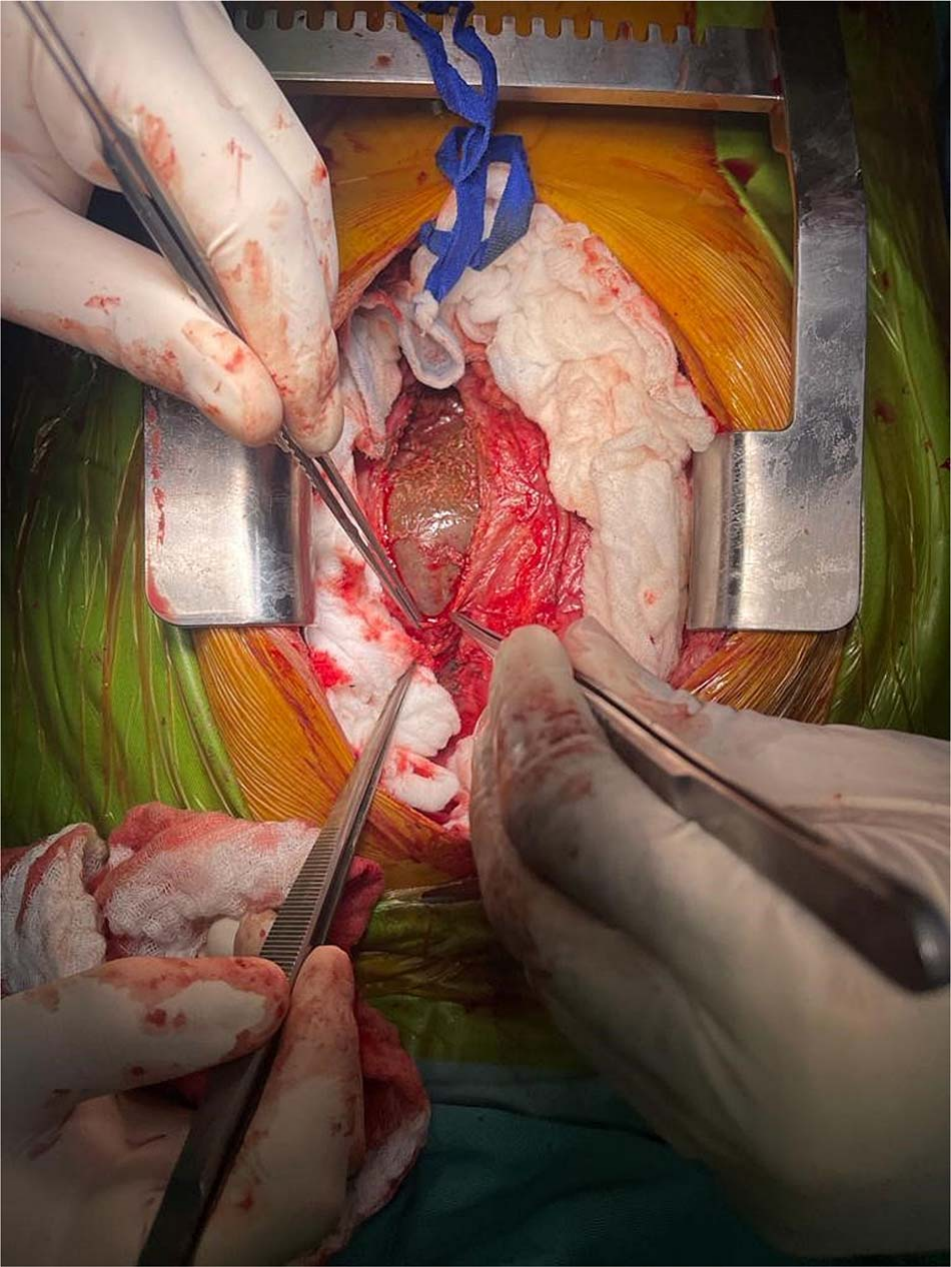
Intraoperative view of the pericardial hydatid cyst.

**Figure 5:**
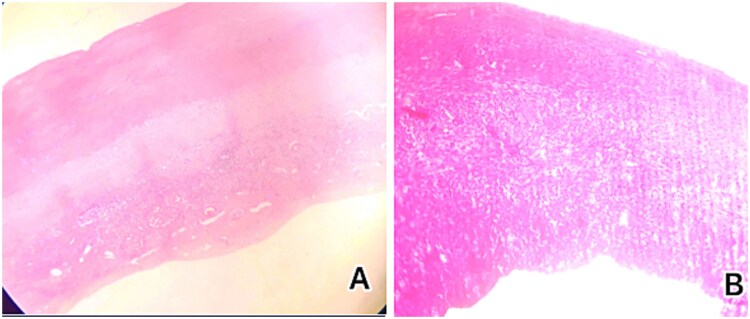
Histological examination showed (A) low-power microscopic image illustrating the outer acellular laminated membrane, a transparent nucleated lining, and the outer fibrotic layer and (B) high power view of the image illustrating the outer acellular laminated membrane and the outer fibrotic layer with granulation tissue with increased eosinophils. Note focal hemorrhage is evident.

A diagnosis of right-sided heart failure due to an intrapericardial mass was made. The cardiothoracic surgery team performed urgent surgery through a small chest incision. During the operation, a large cystic structure measuring ~ 6 by 7 cm was found inside the pericardial sac and was completely removed (Fig. 4). Around 800 cubic centimeters of bloody fluid from the pericardial sac and pleural cavities was drained. Tissue analysis confirmed the diagnosis (Fig. 5).

Following surgery, the patient showed significant improvement. Liver enzyme levels decreased, with alanine aminotransferase at 89 units per L and aspartate aminotransferase at 109 units per L. White blood cell count was 11 000 per μl, and hemoglobin was 12 g/dl. The patient became free of fever and was discharged after 5 days on Albendazole 400 mg twice daily for 2 months.

At a 1-week follow-up, the surgical wound had healed well with no signs of infection. The patient reported significant improvement and was satisfied with the outcome of the surgery.

## Discussion

The hydatid disease, which is primarily found in endemic regions like the Mediterranean Basin, Australia, New Zealand, North Africa, Eastern Europe, the Balkans, the Middle East, and South America, is a dangerous and potentially fatal illness caused by the larvae of the Echinococcus granulosus [4].

Pericardial hydatid cysts are uncommon; they account for 0.5%–2% of hydatid cyst cases and 2%–10% of cases of cardiac hydatidosis. When pulmonary echinococcal cysts rupture, the heart is mostly invaded by the coronary arteries or pulmonary veins, resulting in cardiac involvement [5].

According to a systematic review of 37 studies on cardiac hydatid cysts, cardiac involvement is uncommon and linked to non-specific symptoms like fatigue, palpitations, dyspnea, and chest discomfort [3].

A prompt diagnosis that takes into account both laboratory and radiological findings is essential. Chest radiography results are reliable and include abnormalities of the heart outline, such as deformations or calcifications. An electrocardiogram, a simple, safe, and quick exam, may reveal deep T-wave inversions, arrhythmias, or conduction problems such as heart block.

Echocardiography is the diagnostic method of choice. CT is a reliable method for diagnosing cardio pericardial hydatid cysts, distinguishing solid tumors from watery ones, and searching for other locations. When combined with echocardiography, CT scans provide precise information on abnormality sites [6]. Surgery is the mainstay for cardiac hydatid cysts followed by a course of albendazole therapy according to WHO recommendations.

## Conclusion

Pericardial hydatid cysts are even a rare condition but it is a life-threatening condition, so an early diagnosis is important that prompt early surgical intervention with later appropriated antiparasitic therapy to prevent its severe complications.
